# Wood Consumption by Geoffroyi’s Spider Monkeys and Its Role in Mineral Supplementation

**DOI:** 10.1371/journal.pone.0025070

**Published:** 2011-09-28

**Authors:** Oscar M. Chaves, Kathryn E. Stoner, Sergio Ángeles-Campos, Víctor Arroyo-Rodríguez

**Affiliations:** 1 Pontifícia Universidade Católica do Rio Grande do Sul, Porto Alegre, Rio Grande do Sul, Brazil; 2 Centro de Investigaciones en Ecosistemas, Universidad Nacional Autónoma de México (UNAM), Antigua Carretera a Pátzcuaro No. 8701, Ex Hacienda de San José de la Huerta, Morelia, Michoacán, México; 3 Department of Biological and Health Sciences, Texas A & M University-Kingsville, Kingsville, Texas, United States of America; 4 Departamento de Nutrición Animal y Bioquímica, Facultad de Medicina Veterinaria y Zootecnia, Universidad Nacional Autónoma de México, Delegación Coyoacán, Mexico; University of Florence, Italy

## Abstract

Wood consumption is a rare behavior in frugivorous primates; however, it can be necessary for nutritional balancing as it may provide macro and/or micronutrients that are scarce in the most frequently eaten items (fruits). We tested this hypothesis in six spider monkey (*Ateles geoffroyi*) communities inhabiting continuous and fragmented rainforests in Lacandona, Mexico. We investigated the importance of both live and decayed wood in the diet of the monkeys, and assessed if wood consumption is related to the nutritional composition of these items. In general, wood consumption was focused on trees of *Licania platypus* (Chrysobalanaceae) and *Ficus* spp. (Moraceae), and was similar in continuous forest and in fragments (mean ± SD; 24±20% vs 18±16% of total feeding time, respectively), but marginally higher in females than in males (16±14% vs 5±4%, respectively). Live and decayed wood were both poorer in lipids, proteins, total nonstructural carbohydrates, and total digestible nutrients compared to mature and immature fruits. Moreover, decayed wood of *L. platypus* showed consistently higher levels of sodium and calcium compared to fruits. In conclusion, our findings suggest that wood from decaying trees of *L. platypus* and *Ficus* spp. and young branch piths of *L. platypus* represents an important source of sodium and/or calcium in the diet of spider monkeys, particularly in the case of females. The protection of decaying trees within forests and fragments is therefore necessary for the appropriate management and conservation of this endangered primate species.

## Introduction

Information about qualitative or quantitative mineral requirements of wild nonhuman primates is scarce, but the importance of minerals for all animals is unquestionable [1]. Minerals have multiple functions in organisms̀ bodies, e.g., as structural components of organs and tissues, as cofactors or activators in enzyme and hormone systems, as constituents of body fluids and tissues, and as regulators of cell replication and differentiation [1], [2]. Nevertheless, some minerals, such as sodium, are scarce in tropical soils [3] and most terrestrial plants, and hence, herbivorous primates must supplement their diets with other foods [4], [5], [6]. For example, geophagy (dirt-eating) has been proposed as a strategy to supplement dietary mineral supply in humans [4] and many non-human primates [5]. Similarly, several African primates have been observed feeding on sodium-rich plant species/items to supplement their diets, e.g., lowland gorillas *G. gorilla gorilla* [6], [7], black and white colobus *Colobus guereza*, *Piliocolobus tephrosceles* [8], and chimpanzees *Pan troglodytes* [9].

For most tropical primates, fruits and leaves (and insects in some cases) represent common food items in the diet, satisfying their main nutritional requirements [1], [10], [11], [12]. In contrast, consumption of less digestible plant items, such as decayed wood, branches and tree bark, has been considered less important in their diet because woody items are rarely consumed (<1% of total feeding time, see below). In general terms, these items present a lower content of protein, energy and nonstructural carbohydrates, and a higher content of cellulose and lignin than other foods [7], [10], [13].

Wood consumption by primates often varies from 0 to 3% of total feeding time, e.g., Tonkean macaques *Macaca tonkeana* [14], red howlers *Alouatta seniculus* [15], black howlers *A. caraya* [16], and woolly spider monkeys *Lagothrix lagotricha* [17]. However, a growing number of studies on primates demonstrate that wood consumption may contribute to a noticeable percentage of the diet in some cases, e.g., 4% in *G. beringei* [6], *ca*. 10% in red-handed howlers *Alouatta belzebul* [18], up to 38% in *A. seniculus* [19]. The explanation for this feeding behavior is unclear, but three main nonexclusive hypotheses have been proposed: (i) wood represents a rich source of some essential minerals, such as sodium, which are scarce in other plant items, e.g., *P. troglodytes* [9], *G. beringei* [6], [7], *C. guereza* [20], *C. guereza* and *P. tephrosceles* [8]); (ii) wood is a source of arthropod prey, and hence, protein, e.g., *P. troglodytes* and *G. gorilla* [21]; and (iii) wood has a medicinal effect, e.g., *P. troglodytes* [22]. Because these hypotheses only have been tested for African primates (chimpanzees, gorillas, and colobus monkeys), further studies are needed to explore whether they can be generalized to other habitats and species.

If wood is an important source of minerals and/or macronutrients, wood consumption may be particularly important for primates in forest fragments, where food availability is typically lower than in continuous forests [23], [24], [25]. Additionally, wood consumption may vary between sexes because mineral requirements are often greater in females than males [1]. In fact, females often devote more time feeding (e.g. Peruvian spider monkeys *Ateles chamek* [26]), while males spend more time traveling and defending females against males from neighboring groups, e.g., *P. troglodytes* [27], *Ateles geoffroyi* [28], [29]. However, to date there are no systematic studies evaluating the relative importance of wood in the diet of Neotropical monkeys and its potential role in mineral supplementation, neither in continuous forests nor in forest fragments.

In specialized frugivorous primates, such as *Ateles* spp. [10], [30], the digestive system is designed essentially for a fleshy fruit diet and, therefore, they are presumably physiologically constrained in how much woody and fibrous material they are able to digest [10], [31]. However, throughout their range, *A. geoffroyi* can spend up to 14.2% of time feeding on decayed wood and other fibrous items [32], while in southern Mexico this species invests 23% of the feeding time on decayed and live wood [24]. Here we test if wood consumption by *A. geoffroyi* is related to mineral supplementation. We first analyze the importance of both live and decayed wood in the diet of six *A. geoffroyi* communities inhabiting continuous and fragmented rainforests in Lacandona, Mexico, and test if wood consumption differs between forest conditions, wood types and sexes. Second, we assess if wood consumption is related to the nutritional composition of these items, compared to fruits. Our study suggests that wood consumption by *A. geoffroyi* is related to a high content of sodium and calcium in this material, compared to other food items. Knowledge of how endangered species, such as spider monkeys, meet their nutritional requirements is crucial to improve our understanding of dietary selection, and hence, in assessing habitat suitability and generating appropriate conservation plans.

## Methods

### Study sites and monkey communities

This work was conducted in Lacandona, southern Chiapas, Mexico (16°05'58″ N, 90°52'36″ W). The vegetation in the area is lowland tropical rainforest and semideciduous rainforest [33]. The climate in the region is hot and humid with 23.9°C average temperature and 2881 mm average annual rainfall. The study was conducted in two adjacent areas separated by the Lacantún river: the Marqués de Comillas fragmented region (MCR, eastern side of the river), and the continuous forest of the Montes Azules Biosphere Reserve (MABR, western side). A detailed description of these areas is provided by Chaves et al. [24].

We studied six spider monkey communities ranging in size from 35 to 44 individuals. They were located in three different areas of MABR (hereafter referred as C1, C2, and C3) separated by at least 4 km, and in three forest fragments of MCR (hereafter referred as F1, F2, and F3) located in the communities of Reforma Agraria (1125 ha fragment, 16°15'12.2″N, 90°49'59.5″W) and Zamora Pico de Oro (14.4 and 31 ha fragments, 16°19'24.5″N, 90°50'43.7″W), respectively. All fragments in MCR were isolated ≥24 years ago, are immersed in similar anthropogenic matrices (pastures, cocoa plantations, agricultural lands and rural settlements), and their distances to continuous forest ranged from 200 to 1200 m. For the three study sites of MABR (>300,000 ha) and for the largest fragment, we restricted data collection of feeding behavior to an area of 32–90 ha (depending on the home ranges of each monkey community), whereas for the other two fragments the entire area was sampled [24]. Overall, the diet of the study monkeys comprises 121 plant species belonging to 39 families, and fruits are the most eaten items (i.e. 56% of total feeding time) [24].

### Feeding behavior

Diet of spider monkeys was studied during a 15-mo period (6 months in dry season: February-April 2007 and 2008; and 9 months in rainy season: May-Jun and July-October 2007, and August-October 2008). Feeding behavior was documented for each of the six focal monkey communities during 3 consecutive days once every 3 weeks, using 5-min focal animal sampling of adult individuals of both sexes [34]. During the follows, spider monkeys were sighted with the aid of visual and auditory cues (e.g. vocalizations, rustling tree crowns, and dropping branches or fruits) and high resolution binoculars (Swarovski SLC 10×42). Focal animals were randomly changed at 5-min intervals or when animals moved out of sight. Data were collected from 0700 h to 1730 h, totaling 1010 h of focal observations (496 h in continuous forest and 514 h in fragments), from which 448 h (44%) were feeding observations (205 h in continuous forest and 243 h in fragments). When monkeys were feeding on wood, we recorded the species eaten, considering two categories: live wood (i.e. young branch piths, woody stems, and bark) and decayed wood (i.e. woody pieces removed from trunk of decaying trees). For species exploited for live wood, we also estimated the density of trees ≥10 cm in diameter at breast height in 10 2×50 m linear transects by study site . See Chaves et al. [24] for further details about differences in vegetation composition and structure among sites.

The relative importance of different plant species exploited for live or decayed wood was calculated as the percentage of time spent consuming a particular species in relation to the total feeding time on all species and plant items. Following Rode et al. [8], we assumed the percent of time spent on wood to be related to the percent contribution of the wood to total dry matter intake. As an indicator of the degree to which spider monkeys are selective in their choice of food tree species exploited for live wood, we used the *W_i_* selection index [35]. This index was calculated as feeding time for each tree species divided by the density of the species in our plots. A selection index value above 1.0 indicates preference; values less than 1.0 indicate avoidance [35]. Plant nomenclature followed the Royal Botanical Garden and Missouri Botanical Garden update database (http://www.theplantlist.org/, accessed at July 2011).

### Collection of material for nutritional analyses

We collected material in November-December 2008, April 2009, and October 2009. Based on our data of wood consumption (see Table 1), we collected random samples within the tree of decayed wood in four trees of *Licania platypus* located in continuous forest (sites C1 and C2), one tree of *L. platypus* located in fragment F3, and three trees of *Ficus* spp. located in fragments F1 and F2. In each case, we collected at least 1-kg in humid weight. Since wood nutrient content can vary among species [6], [8], [9], we analyzed separately the nutrient content of decayed wood of *L. platypus* and *Ficus*. Similarly, we collected *ca*. 1-kg of *L. platypus* young branch piths from 15 trees from continuous forest and 11 trees from fragments. Although spider monkeys consumed live wood from 11-12 different plant species, we only analyzed samples from *L. platypus* because the consumption of this plant item was largely focused on this species in continuous forest and fragments (76% and 67% of time feeding on live wood, respectively). Moreover, most plant species exploited for live wood were large tree species and/or canopy vines (see Results), which strongly limited our access to samples. Finally, we collected *ca*. 0.3-kg of ripe fruit pulp from four top fruit species in the diet of *A. geoffroyi* [24] available during the sampling period. These species were: *Ficus tecolutensis* (Moraceae), *Spondias radlkoferi* and *S. mombin* (Anacardiaceae), and *Sabal mexicana* (Arecaceae); and one additional food species (*Attalea butyracea*, Arecaceae) with lower importance in the diet of spider monkeys at our sites (i.e. <0.7% of total feeding time) [24]. Since mineral concentrations within a tree can be affected by local soil mineral availability [36] and this can influence the foraging behavior of tropical primates [37], we collected the samples from trees used by *A. geoffroyi* during the study period. In the laboratory, samples were weighed shortly after collection and then were dried in a drying oven (40–50°C), and later transported to the Laboratorio de Nutrición Animal y Bioquímica, Universidad Nacional Autónoma de México (UNAM), for nutritional analyses. Immediately before chemical analysis, a portion (15–20%) of the previously dried samples was dried again at 100°C to remove any absorbed atmospheric water to calculate the true dry matter of each sample.

**Table 1:** Plant species exploited for live and decayed wood by spider monkeys (*Ateles geoffroyi*) in continuous forest and forest fragments in the Lacandona rainforest, Mexico.

Species	Family	GF	Mean % of total feeding time			Density (stems/3000 m^2^)^a^	*W_i_* b
			Total	Decayed	Live		
Continuous forest							
*Licania platypus* (Hemsl.) Fritsch	Chrysobalanaceae	Tree	28.7	19.9 (0.03,28,32)	8.8 (0.3,9,16)	2	24
*Strychnos tabascana* Seem.	Loganiaceae	Vine	1.1	0	1.1 (0,0.2,3)	―	―
*Swietenia humilis* Zucc.	Meliaceae	Tree	0.7	0	0.7 (0.1,0.8,1)	2	1.9
vines (4 morphospecies)	—	Vine	0.55	0	0.34 (0.1,0.2,0.7)	―	―
*Spondias radlkoferi* Donn.Sm.	Anacardiaceae	Tree	0.3	0	0.3 (0,0.02,0.9)	5	0.31
*Nectandra reticulata* Mez	Lauraceae	Tree	0.1	0	0.1 (0,0,0.3)	4	0.13
*Pouteria campechiana* (Kunth) Baehni	Sapotaceae	Tree	0.1	0	0.1 (0,0,0.4)	7	0.08
*Brosimum alicastrum* Sw.	Moracaeae	Tree	0.03	0	0.03 (0,0,0.1)	5	0.03
*Guarea glabra* Vahl	Meliaceae	Tree	0.03	0	0.03 (0,0,0.1)	27	0.01
*Hirtella americana* L.	Chrysobalanaceae	Tree	0.02	0	0.02 (0,0,0.05)	1	0.11
*Lonchocarupus* sp.	Fabaceae	Tree	0.02	0	0.02 (0,0,0.06)	1	0.11
*Luehea seemannii* Triana & Planch	Malvaceae	Tree	0.02	0	0.02 (0,0,0.06)	2	0.05
Total			**31.5**	**19.9**	**11.6**	**52**	
Fragments							
*Ficus* spp. (2 spp.)	Moracaeae	Tree	10	10.0 (0,8,22)	0	―	
*Licania platypus* (Hemsl.) Fritsch	Chrysobalanaceae	Tree	3.3	1.3 (0,0.6,3)	2.0 (0,2,4)	6	3.9
vines (3 morphospecies)	—	Vine	0.37	0	0.4 (0,0.4,0.7)	―	
*Blepharidium guatemalense* Standl.	Rubiaceae	Tree	0.3	0	0.3 (0,0,0.8)	3	1.18
*Guarea guidonia* (L.) Sleumer	Meliaceae	Tree	0.2	0	0.2 (0,0,0.6)	8	0.3
*Mouriri myrtilloides* (Sw.) Poir.	Melastomataceae	Tree	0.07	0	0.07 (0,0,0.2)	2	0.41
*Psychotria* sp.	Rubiaceae	Shrub	0.03	0	0.03 (0,0,0.1)	1	0.36
*Castilla elastica* Cerv.	Moracaeae	Tree	0.02	0	0.02 (0,0,0.07)	4	0.06
*Cupania dentata* Moç. & Sessé ex DC.	Sapindaceae	Tree	0.02	0	0.02 (0,0,0.07)	1	0.24
*Luehea seemannii* Triana & Planch	Malvaceae	Tree	0.02	0	0.02 (0,0,0.05)	2	0.11
*Spondias radlkoferi* Donn.Sm.	Anacardiaceae	Tree	0.02	0	0.02 (0,0,0.06)	5	0.05
*Posoqueria latifolia* (Rudge) Schult.	Rubiaceae	Tree	0.01	0	0.01 (0,0,0.03)	0	119
Total			**14.4**	**10.7**	**3.01**	**32**	

Species are listed by order of importance in the diet.

a Only trees with ≥10 cm in diameter at breast height were considered (see further details in Chaves et al [24]). Although dead standing trees were not observed during the vegetation samplings, our observations through the home ranges of each monkey group indicated that their density was *ca.* 0.03 trunks/10,000 m^2^ in both habitat types.

b Index of preference. See Methods.

Plant growth form (GF) and average percentage of total feeding time (%TFT). The %TFT for the three study sites is indicated in parenthesis.

### Nutritional analyses

Ether extracts (primarily lipids) were determined with the Soxhlet method [38]. Percent of ether extract was calculated as the weight difference between the original sample and the fat-free sample. Crude fiber was obtained from the remnants left after boiling the sample in acid and alkali with the modified method of Heneberg-Stohmann [38], and crude ash was obtained after incineration at 550°C. The value of nitrogen-free extract (i.e. total nonstructural carbohydrate, TNC) was estimated by subtracting the weights of the above components from the total sample dry weight [38]. To determine the percentage of fiber, we first analyzed the samples for neutral detergent fiber (NDF) and acid detergent fiber using (ADF) an Ankom 200 Fiber Analyzer (Ankom, Inc., Macedon, NY) [38]. Hence, following Helrich [38], we determined the percentage of cell wall composed by cellulose, hemicellulose, and lignin by the digestion of the sample (including NDF and ADF). The percentage of hemicellulose and lignin was calculated from the ADF fraction. Finally, we calculated the percentage of hemicellulose by subtracting the percentages of cellulose and lignin from the NDF fraction.

Total nitrogen (N) in each sample was determined by the Kjeldahl method and multiplied by the 6.25 conversion factor to estimate crude protein content [39], [40]. Although some uncertainty exists whether 6.25 is the best conversion factor for tropical fruits [38], we use it here to allow for comparison with other similar studies. Despite that total N is considered a poor indicator of available protein in diet of primates [41], it is a simple, economic, and widely used method to estimate crude protein in primate diets [40], [42]. As an indicator of the percentage of energy digestible in each wood and fruit type we estimated the total digestible nutrients (TDN) using the formula: TDN  =  % of digestible crude protein + % of TNC + % of digestible crude fiber + (% of digestible lipids ×2.25).

Calcium content was quantified using the laboratory protocol described by Helrich [38]. We also analyzed the content of five other important minerals (Na, Mg, Fe, P, and Zn) using a Perkin Elmer AAS-800 atomic absorption spectrophotometer (Wellesley, MA). Methods for mineral tests are explained in more detail by Helrich [38].

### Data analysis

We used generalized linear models (GLMs) to test the effects of forest condition (continuous and fragmented), wood type (live and decayed), sex (male and female), and the interaction between them on proportion of time feeding on wood. We used an arcsine transformation to normalize the proportion data, and selected a normal distribution with an identity link-function for the response variables [43]. To identify which treatments were statistically different between each other we used post-hoc analyses with contrasts [43]. We used the three sites per forest condition as replicates. We also used GLMs to test for differences in macronutrients and micronutrients between plant items (decayed wood, live wood, mature fruits and immature fruits). Data of nutrient content were normalized using arcsine transformations (for macronutrients) and log-transformations (for micronutrients). The whole models were: CONTENT of each macronutrient or micronutrient  =  NUTRIENT nested in PLANT ITEM + PLANT ITEM. When we found differences among plant items in macronutrients or micronutrients, we used contrast tests to identify which plant item differed from each other [43]. All statistical analyses were performed using JMP software (version 8.0, SAS Institute, Cary, N.C.).

### Ethics statement

All research reported in this study adhered to the laws of the Mexican Government (SEMARNAT, Secretaría de Medio Ambiente y Recursos Naturales; PROFEPA, Procuraduría Federal de Protección al Medio Ambiente) to work with wild animals in Lacandona, and the recommendations of the Weatherall report, “The use of nonhuman primates in research”. Since our work is not invasive, only observational, we meet all ethical and legal requirements established by the American Society of Primatologists (ASP), Animal Care and Use Committee, and Ethical Committee of the Zoological Society of London for work on primates. Although our institution, Universidad Nacional Autónoma de Mexico (UNAM), does not yet have an IRB or a similar governing body of ethics, this project was approved by the institutional authorities from UNAM and Consejo Nacional de Ciencia y Tecnología (project CB-2006-56799). We thank the Comisión Nacional de Áreas Naturales Protegidas (CONANP) of Mexico and the owners of the forest fragments for giving us the permission to perform the research in the study sites.

## Results

### Wood Consumption

**Figure 1:**
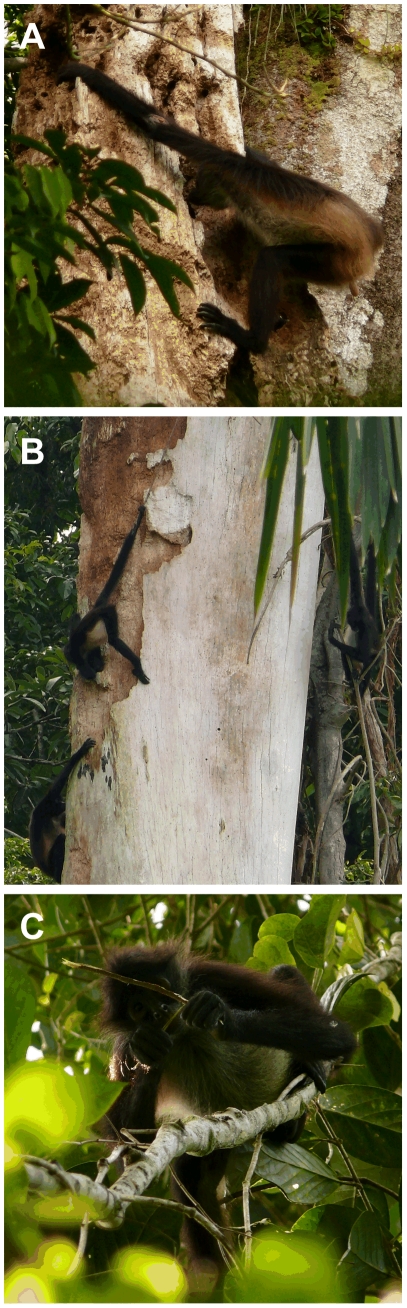
Spider monkeys eating wood in Lacandona rainforest, Mexico. Panels show consumption of decayed wood from *Licania platypus* (A) and *Ficus* sp. (B), and live wood from young branches of *L. platypus* (C).

Overall, we collected 1568 5-min records of wood consumption (1041 records from decayed wood and 527 records from live wood), representing 107 h (75.2 h in decayed wood and 31.8 h in live wood). In continuous forest, consumption of decayed wood was restricted to five decaying trees of *Licania platypus* (20% of total feeding time, TFT, Table 1), while in fragments the consumption of this food item was focused in three decaying trees of *Ficus* spp. and one decaying tree of *L. platypus* (10 and 0.7% of TFT, respectively, Table 1). On several occasions, we observed that spider monkeys returned to the same decaying tree to feed on consecutive days, and spent 0–5.2 h/day surrounding the tree as individuals took turns feeding. During these feeding bouts, monkeys either chewed the wood directly from the decaying tree while sustaining themselves with their arms, legs and tails (Figure 1A,B), or removed pieces of approximately 5–25 cm in length and moved to a nearby tree to eat them.

In both habitat types the consumption of live wood was focused on young branch piths of a diverse plant assemblage: 11 species (and 4 morphospecies of vines) in continuous forest and 12 species (and 3 morphospecies of vines) in fragments (Table 1). In most cases the %TFT was proportional to the density of each species in the study area (Table 1), however, in both habitats, spider monkeys showed a clear preference for the live wood of *Licania platypus* (i.e. the most exploited species in both habitat types, Table 1). Four tree species (*L. platypus*, *Spondias radlkoferi*, *Luehea seemannii*, and *Guarea guidonia*) consumed for live wood were found in both habitat types (Table 1). In spite of this diversity, in both continuous forest and fragments most consumption of live wood was focused on young branch piths of *L. platypus* (8.8% and 2% of TFT, respectively, Table 1). In all cases, spider monkeys used their hands and teeth to break the young branches (*ca*. 25–60 cm in length) and to extract their piths (Figure 1C), immediately dropping branches afterwards.

### Influence of forest condition, wood type, and sexes on wood consumption

Wood consumption was highly variable among sites, wood types, and sexes. In continuous forest, wood consumption varied from 0.8% to 36.5% of TFT (mean±SD; 23.8±19.9%); whereas in fragments, it averaged 18.1 (± 16.2%), ranging from 6.4% to 36.6% of TFT. As consequence, we did not find significant differences in wood consumption between forest conditions (FOREST; *χ* ^2^  =  0.12, df  =  1, *P*  =  0.73). Wood consumption was also similar when comparing decayed wood (15.1±14.3%) versus live wood (5.8±5.3%) (WOOD TYPE; *χ* ^2^  =  2.7, df  =  1, *P*  =  0.10). Wood consumption was higher in females (15.8±14.0%) than in males (5.2±3.6%), but this pattern was only marginally significant (SEX; *χ* ^2^  =  3.5, df  =  1, *P*  =  0.06). Finally, none of the interacting factors were significant (FOREST × WOOD TYPE, *P*  =  0.59; FOREST × SEX, *P*  =  0.33; WOOD TYPE × SEX, *P*  =  0.78; FOREST × WOOD TYPE × SEX, *P*  =  0.69).

### Nutritional composition of wood and fruits

**Figure 2:**
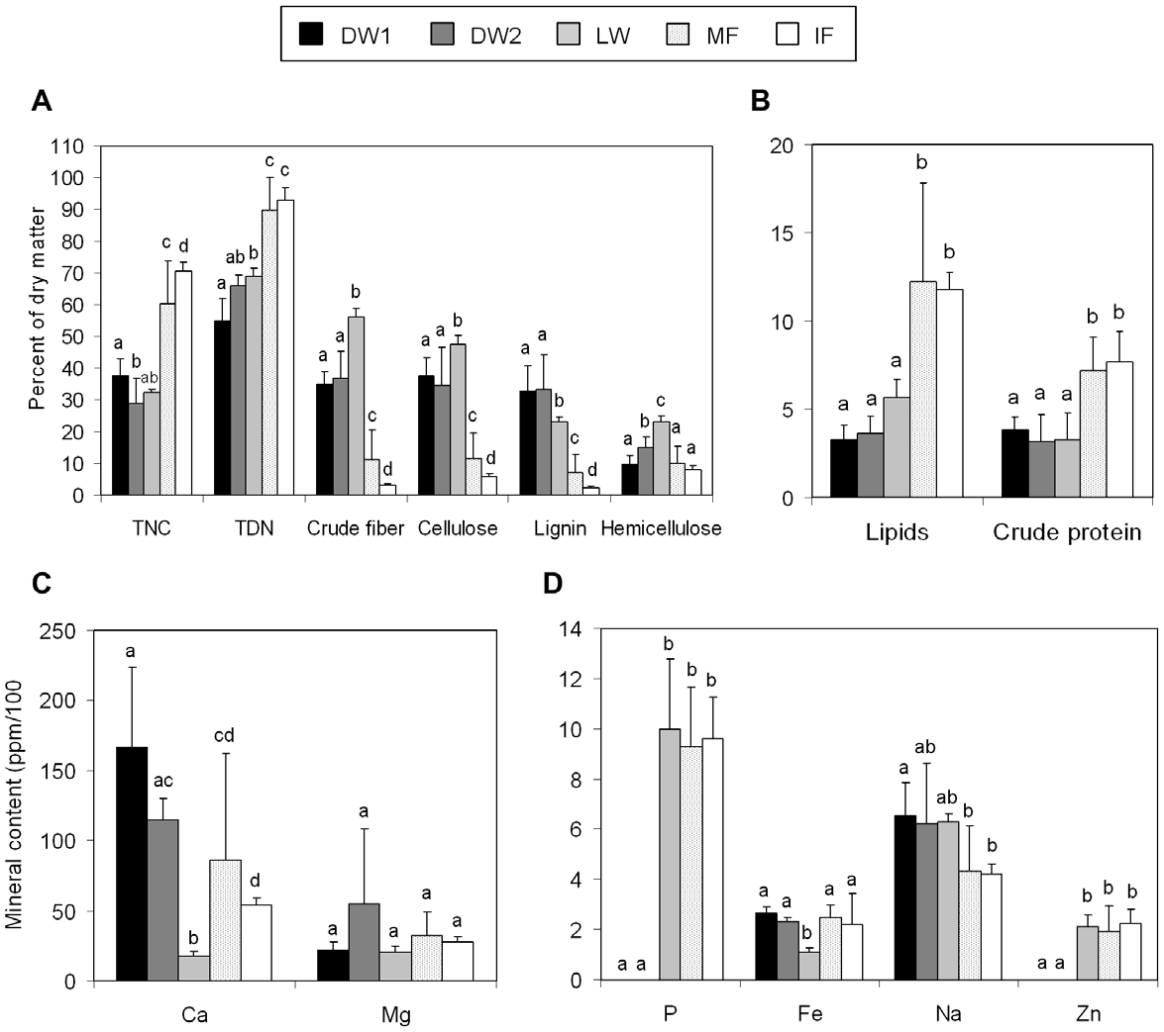
Nutritional composition of plant food species. Average (±SD) content of macronutrients (A,B) and minerals (C,D) of different plant items exploited by spider monkeys in Lacandona rainforest, Mexico. In each figure, nutrients are listed in decreasing order (from left to right). DW1 =  decayed wood of *L. platypus*; DW2 =  decayed wood of *Ficus* spp.; LW =  live wood; MF =  mature fruits; IF =  immature fruits. Bars sharing a letter are not significantly different (contrast tests, *P*>0.05).

Overall, macronutrient concentrations varied greatly among species and plant items, particularly between wood items and fruits (Table 2, Figure 2). Percent of macronutrients differed significantly among plant items (*χ* ^2^  =  725, df  =  39, *P*<0.0001), being decayed wood and live wood lower in content of TNC, TDN, lipids, and crude protein compared to mature and immature fruits (Figure 2A,B). By contrast, percent of crude fiber was higher in live wood, followed by decayed wood, mature fruits and immature fruits (contrast tests, *P*<0.05 in all cases, Figure 2A). The percent of lignin was higher in decayed wood, followed by live wood, mature fruits and immature fruits (contrast tests, *P*<0.05 in all cases, Figure 2A). The percent of cellulose and hemicellulose were higher in live wood and decayed wood of *Ficus* than in the other plant items (*P*<0.05 in all cases, Figure 2A).

**Table 2:** Average (± SD) content of macronutrients of different plant items in eight food species for spider monkeys (*Ateles geoffroyi*) in the Lacandona rainforest, Mexico.

Plant species	Item^b^	*n*	Cellular content				Cell wall			
			CC	Lipids	CP	TNC	CF	CE	HC	Lignin
*Licania platipus* (Chrysobalanaceae)	LW	8	6.2 (2.2)	5.6 (1)	3 (1.6)	31.8 (1.2)	56.2 (2.4)	47.6 (2.8)	23 (1.9)	23.1 (1.6)
	DW	8	20.3 (8.2)	3.2 (0.9)	3.9 (0.7)	37.4 (5.4)	34.9 (3.7)	37.4 (5.9)	9.5 (3.2)	32.7 (7.9)
*Ficus* spp. (2 spp) (Moraceae)	DW	4	16.7 (6.5)	3.6 (1.0)	3.2 (1.5)	28.9 (8.1)	36.9 (8.2)	34.7 (11.7)	15.2 (3.2)	33.3 (11.1)
*F. tecolutensis* (Moraceae)	RF	3	45.7 (9.3)	11.3 (1.2)	7.9 (0.9)	51.5 (7.2)	18.6 (2.1)	25.7 (8.9)	10.4 (2.8)	17.9 (1.3)
*Attalea butyracea* (Arecaceae)^a^	RF	1	—	25	7.3	38.6	17.9	—	—	—
*Sabal mexicana* (Arecaceae)	RF	4	49.3 (8.3)	5.2 (1.2)	4.8 (1.1)	43.2 (8.4)	28.3 (2.7)	20.8 (7.3)	18.9 (2.7)	10.9 (1.1)
*Spondias radlkoferi* (Anacardiaceae)	RF	5	80.8 (5.6)	11.2 (0.9)	7.5 (2.1)	69.6 (1.8)	4.8 (1.0)	6.9 (0.5)	8.2 (4.4)	4.1 (1.8)
	UF	1	81.4	12	5.2	73	3.4	6.9	9.6	2
*S. mombin* (Anacardiaceae)	UF	3	84.9 (0.9)	11.7 (1.2)	8.5 (0.9)	69.7 (2.8)	2.9 (0.3)	5.2 (0.5)	7.4 (0.8)	2.4 (0.6)

a With exception of this palm species, the rest of plant species represent “top” food species in the diet of spider monkeys in Lacandona [24].

b LW  =  live wood, DW  =  decayed wood, RF  =  ripe fruit, UF  =  unripe fruit.

Column headings: *n*  =  number of analyzed samples (see Methods), CC  =  estimated cellular content, CP  =  crude protein, TNC  =  total nonstructural carbohydrates, CF  =  crude fiber, CE  =  cellulose, HC  =  hemicellulose.

Mineral content varied significantly among plant items (*χ* ^2^  =  550, df  =  34, *P*<0.0001; Table 3, Figure 2C,D). Calcium content was similar in decayed wood of *L. platypus* and *Ficus* spp., but higher in decayed wood of *L. platypus* than in live wood and fruits (*P*<0.01 in all cases, Figure 2C). Decayed wood of *Ficus* showed a higher content of calcium than in live wood and immature fruits (*P*<0.05 in both cases) but did not differ from mature fruits (*P*>0.05). Live wood showed the lowest calcium content of all plant items tested (*P*<0.0001 in all cases, Figure 2C). Although a large variation was observed in magnesium content in decayed wood of *Ficus*, we did not detect significant differences among plant items (*P*>0.05 in all cases, Figure 2C). Both decayed and live wood showed a similar content of sodium (*P*>0.05 in all cases, Figure 2D), but only the decayed wood of *L. platypus* showed a higher sodium content than fruit (*P*<0.05 in all cases; Figure 2D). Iron content was lower in live wood than in the other items (*P*<0.05 in all cases; Figure 2D), but no other difference were detected (Figure 2D). Finally, in contrast with most of the items analyzed, phosphorous and zinc were absent in decayed wood, but had similar concentrations in live wood and fruits (Figure 2D).

**Table 3:** Average (± SD) content of minerals of different plant items in seven food species for spider monkeys (*Ateles geoffroyi*) in the Lacandona rainforest, Mexico.

Plant species	Item^a^	*n*	Ca	Mg	Na	Fe	P	Zn
*Licania platipus* (Chrysobalanaceae)	LW	8	1800 (300)	2080 (432)	631.2 (30.7)	112.1 (15.2)	1000 (300)	213.8 (45.6)
	DW	8	17000 (600)	2213.3 (558.6)	656.1 (292.4)	265.5 (23.7)	—	—
*Ficus* spp. (2 spp) (Moraceae)	DW	4	11000 (100)	5478.4 (5358)	622.7 (240.4)	233.3 (14.4)	—	—
*F. tecolutensis* (Moraceae)	RF	3	5000 (200)	6500 (477)	390.1 (15.9)	173.3 (15.1)	500 (33)	250 (28)
*Sabal mexicana* (Arecaceae)	RF	3	11000 (200)	960 (124)	912.2 (56.1)	250.0 (21.4)	—	—
*Spondias radlkoferi* (Anacardiaceae)	RF	5	5000 (1000)	3060 (493)	344.4 (49.4)	266.6 (42.1)	1000 (200)	221.2 (67.3)
	UF	1	5000	2400	471.7	133.3	1000	156.2
*S. mombin* (Anacardiaceae)	UF	3	6000 (400)	2933.3 (305.5)	403.6 (32.1)	248.9 (131.6)	1000 (100)	247.9 (42.5)

a LW  =  live wood, DW  =  decayed wood, RF  =  ripe fruit, UF  =  unripe fruit.

## Discussion

Overall, our findings suggest that wood contributed to an important part of the diet of spider monkeys in both continuous forest and fragments, and support the idea that wood represents a source of some minerals (i.e. sodium and calcium) which are less abundant in fruits. This implies that wood-feeding behavior in some populations of spider monkeys may be necessary to achieve an appropriate nutritional balance. By eating a variety of different plant items (including live and decayed wood) from a diverse species assemblage [30], [32] it is more likely that spider monkeys will obtain an optimal level of each micronutrient required, as has been proposed for different wild primates [1], [12].

Decayed wood came principally from only a few trees of *Licania platypus* and *Ficus* spp. Although most studies on the diet of spider monkeys do not report wood consumption [30], we suggest that it is because of the logistical difficulties of observing this behavior in wild populations (e.g. it requires exhaustive follows of the community through the day) and/or because wood is a negligible food item in the diet, and hence, it is often included in a more general food category, e.g,. typically named “other” [26], [30].

Our findings concur with some previous studies showing that wood can be an important item in the diet of some populations of *Ateles* spp. For instance, decayed wood from a single unidentified rotten tree represented *ca*. 9% of the diet of a group of *A. belzebuth* over a 10-mo study in La Macarena, Colombia [44]. Similarly, in Yasuní, Ecuador, decayed wood from a small number of dead trees was the second most important food item in the diet (i.e. 9.6% of diet) of this same species during a 1-yr study [45]. In the latter forest, Di Fiore et al. [30] report that during a 1-yr period decayed wood accounted for 3.6% of the spider monkeys’ feeding time and, as we also found in Lacandona, spider monkeys frequently visited the same tree and spent large amounts of time feeding on it. In a population of *A. hybridus* living in a fragmented forest in San Juan del Carare, Colombia, wood consumption accounted for up to 37% of total feeding time [19]. Finally, in Voltzberg, Surinam, *A. paniscus* consumed an indeterminate amount of wood from at least 8 plant genera: *Licania* sp., *Dimorphandra*, *Inga*, *Pithecellobium*, *Sacoglottis*, *Nectandra*, *Couratari*, and *Quassia* [30]. This large variation in wood consumption among populations of spider monkeys could be related to differences in mineral content of the fruit species (and/or wood types) available in each study site. Further studies are necessary to assess this hypothesis.

The large amount of time devoted by spider monkeys to wood consumption appears paradoxical because spider monkeys’ digestive tract is presumably specialized for a highly frugivorous diet and, therefore, the quantity of woody material they can process is constrained [10], [31]. In concurrence with other studies reporting wood-feeding behavior in tropical primates [6], [7], [9], we found that although live and decayed wood are poor quality foods (i.e. they are poorer in lipids, proteins, total nonstructural carbohydrates, and total digestible nutrients, and present a higher fiber content than fruits), in general, they showed high sodium content. In particular, the decayed wood of *L. platypus* contained higher concentrations of sodium than fruits (Fig. 2D). This suggests that spider monkeys could be investing a considerable percent of their feeding time on wood consumption because it is an important source of sodium. Similar findings also have been reported for bark consumption in small rodents [46] and arboreal marsupials [47].

In contrast to some other studies that report a lower content of calcium in decayed wood than in fruits and other plant items [6], [9], we found that decayed wood of *L. platypus* was also richer in calcium than the other plant items (Fig. 2C). Thus, our study suggests that wood consumption by *Ateles geoffroyi* has an important role in mineral supplementation. Consistent with this idea, some studies report that to obtain an appropriate mineral balance in the diet, spider monkeys can feed on soil [48], [49], [50]. Similarly, in Kibale, western Uganda, Oates [51] reports that *Colobus guereza* come to the ground and wade through water to forage on swamp plants with high sodium concentrations. In addition, urine consumption is a common behavior in *Cercopithecus ascanius* to deal with a diet deficient in sodium [52].

We cannot confirm that wood consumption is a direct result of the scarcity of sodium and calcium content in fruits (i.e. because we only analyzed five fruit species). Nevertheless, our data strongly suggest that woody material plays a role in mineral supplementation. This role may be particularly important given that, in contrast to most tropical fruits that are only seasonally available [53], decayed wood can be available and exploited by monkeys year around.

Interestingly, we found that females tended to spend more time feeding on wood than males. This suggests that mineral supplementation may be particularly important for females. Minerals such as Na and Ca are critical for reproduction, physiological function and growth in vertebrates [1], [54], [55]. For humans, and presumably nonhuman primates, Ca is one of the most important limiting resources for pregnant [56] and especially lactating females [1], [57], in which a deficiency of Ca can result in health problems such as osteoporosis and osteoarthritis [1]. Unfortunately, specific Na and/or Ca needs of pregnant and lactating spider monkeys are unknown. However, previous studies on gorillas indicate that in adult females the daily intake of these minerals is *ca.* twice that of adult males, while in pregnant women it is *ca.* 20% higher than in adult men [41]. However, further studies including Na and Ca requirements of adult spider monkeys in different phases of reproduction are necessary to understand why females tended to spend more time feeding on decayed wood than males.

Some studies in African primates conclude that wood-feeding behavior is an important strategy for obtaining animal protein in the form of invertebrates [21]. In contrast, we found no evidence in our study that wood-feeding behavior was related to the acquisition of animal protein in spider monkeys. First, the content of crude protein and lipids in the wood was noticeably lower than in fruits (less than 5% of dry matter; Figure 2B). Second, we did not observe arthropods (adults, larvae, or eggs) in the specific sites in which spider monkeys removed pieces of decayed wood, nor in the pieces of wood that we collected for nutritional analyses. The possibility of some medicinal value of wood-feeding in spider monkeys cannot be ruled out until further studies evaluate this hypothesis. Nevertheless, the evidence from our study strongly suggests the value of wood-feeding for obtaining important minerals, namely sodium and calcium.

Overall, as a modest contribution to the understanding of the feeding ecology of spider monkeys, our study suggests that wood from decaying trees of *L. platypus* and *Ficus* spp. and young branch piths of *L. platypus* may represent important sources of sodium and/or calcium in the diet of spider monkey populations in continuous forest and fragments in Lacandona, Mexico. The study is unique in that we studied multiple spider monkey communities in the context of wood-feeding behavior in both continuous forest and fragments.

However, our study has some important limitations that should be addressed in future research to improve our understanding of the role of mineral supplementation in spider monkeys. Specifically, the nutritional analyses were carried out only for a small number of top fruit species and we were unable to measure the mineral intake of fruits or wood for individual monkeys. We did not evaluate the possible influence of age-category on wood consumption or the consequences of wood consumption on individual health. Moreover, available N/protein may be a more ecologically and physiological relevant measure of protein intake by frugivorous animals than crude protein. This is because nitrogen may be bound to lignified plant cell walls or bound to secondary metabolites [40], [41] and because the amount of crude protein that is available in the diet of primates can vary largely among plant items, e.g., it is commonly higher in fruits than in woody items [41]. Therefore, studies including digestibility assays are necessary to obtain a more exact estimation of the protein content of fruits and wood. Similarly, the bioavailability of micronutrients in the diet of spider monkeys (and other primates) needs to be assessed in future studies. This is because it is unclear if the level of micronutrients found in food items is proportionally absorbed by the body, especially when we take into consideration the complex interactions between different micronutrients [59].

Finally, as we mentioned above, the key role of wood in mineral supplementation may depend on site specific differences in spider monkeys' diet, fruit availability, as well as mineral content of fruits. The management of populations of spider monkeys in both forest and fragmented landscapes should take into consideration the protection of all decaying trees of the species recognized in our study. In addition to improving habitat for spider monkeys, this management practice would have positive effects on other wood-depending biota in the forest, e.g., hole-nesting birds [58], thus increasing the biodiversity value (or at least maintaining it) of the managed forests. Furthermore, because the main food items in the diet of some tropical primates appear to be deficient in sodium and other key minerals [1], [8], future research on primate mineral nutrition and digestive physiology will be important to improve our understanding of how primates deal with this limitation.
